# Evaluating the impacts of site preparation and fragment size on coral outplanting success at a highly degraded reef site

**DOI:** 10.7717/peerj.21725

**Published:** 2026-09-14

**Authors:** Yi Da Jiang, Emma F. Camp, Kathryn A. Cobleigh, Christine D. Roper, Paige Strudwick

**Affiliations:** Climate Change Cluster, Faculty of Science, University of Technology Sydney, Ultimo, New South Wales, Australia

**Keywords:** Corals, Coral reefs, Reef restoration, Great barrier reef, Outplanting

## Abstract

Coral reefs are deteriorating worldwide from the combined pressures of global climate change and local stressors. As such, reef restoration interventions are being applied to boost natural recovery and reef health. Outplanting success reported for restoration activities to date has been highly variable. This study evaluated factors that influence outplanting success at an impacted reef, including site preparation (cleaning substrate *vs* uncleaned) and outplant size for two coral species—*Pocillopora acuta* (branching) and *Porites lutea* (massive). To assess overall success, survivorship, health and growth of the two coral species were evaluated over a 170-day period. Survivorship differed between coral species, with *P. lutea* exhibiting higher survival probabilities than *P. acuta* (48.4 ± 7% and 13.6 ± 4% respectively). Low survivorship was largely driven by high dislodgement of coral outplants over the study period (39.58% to 70.83% of outplants dislodged for *P. acuta* and *P. lutea* respectively). Site preparation did not improve coral survival or health and all outplants exhibited algal growth at the end of the study. *P. acuta* outplants exhibited paling prior to *P. lutea* outplants, however, at the end of the study, all outplants except for medium (5–10 cm) *P. acuta* outplants exhibited similar levels of paling. Outplant size did not impact growth or survivorship. Overall, *P. lutea* showed the greatest suitability to outplanting at a degraded site with the highest survival and lowest dislodgement rates. In this study, high dislodgement rates limited outplanting success, indicating that alternative attachment methods and predator deterrents should be evaluated as a priority when using coral outplanting at heavily impacted reef sites. Results from this study demonstrate that not all species are equally suited to outplanting in impacted systems, and highlights the importance of matching species selection to site conditions to optimise restoration outcomes.

## Introduction

In the last decade, coral reef declines have accelerated due to the combined stressors from acute climate change and chronic local pressures (*e.g.*, pollution, overfishing and disease; (Hughes et al., 2017; Hughes et al., 2018; Ward & Myers, 2005; Zaneveld et al., 2016). While mass coral bleaching events driven by marine heat waves are at the forefront of reef declines, chronic local stressors continue to critically undermine reef resilience, with extreme weather events such as tropical cyclones adding substantial additional pressure (Trapp & Hoogewind, 2016). Tropical cyclones can physically and physiologically degrade reef systems through damaging wave action, decreased salinity from extensive rainfall (Jokiel, 2006; Puotinen et al., 2020) or transporting terrestrial pollution offshore (De’ath & Fabricius, 2010). Freshwater discharge into reef systems can cause widespread coral mortality, particularly in inshore reefs, and along shallow reef flats (Edmunds & Gray, 2014; Lough, Lewis & Cantin, 2015; Rodgers et al., 2021). This leads to increases in macroalgae, turfing algae and other benthic organisms (*e.g.*, sponges), and induces phase shifts in the benthic community away from a coral-dominant state which further hinders natural coral recovery (Bruno et al., 2009; Ceccarelli et al., 2020; Graham et al., 2013; Hughes, 1994; Norstrom et al., 2009). Such phase shifts reduce biodiversity, degrade ecosystem functioning and often render coral reefs incapable of natural recovery (Folke et al., 2004; Johns et al., 2018; Norstrom et al., 2009).

Reef restoration efforts that harness sexual or asexual reproduction of corals enhance natural recovery of degraded reefs and show potential to improve resilience to future disturbances (Hein et al., 2021; Suggett et al., 2024). Such approaches are applied in parallel to efforts to mitigate climate change and reduce local stressors (Kleypas et al., 2021; Suggett et al., 2024). The majority of approaches have focused on target coral species that have been most affected by stress events, are important reef-building taxa, quickly restore habitat functions, stabilise substrate and/or provide three-dimensional complexity such as branching and bushy pioneer species including *Acropora* spp. (Schopmeyer et al., 2017; Young, Schopmeyer & Lirman, 2012) and *Pocillopora* spp. (Bostrom-Einarsson et al., 2020; Gardner et al., 2003; Rinkevich, 2000; Yap & Molina, 2003). These coral genera are also suited to in-water asexual propagation and outplanting (*i.e.,* attaching fragments to the reef from a coral nursery or from a donor colony) as they fragment readily, have fast growth rates in coral nurseries and rapidly increase coral cover after outplanting (Rinkevich, 2000). Unfortunately, some corals with fast growth traits show increased susceptibility to thermal stress due to the energetic demands of rapid growth reducing thermotolerance (Wall et al., 2018) and are less successful in highly degraded systems (Darling et al., 2012; Darling, McClanahan & Cote, 2013). Although early restoration guidelines emphasize the importance of diversifying coral taxa (Edwards & Gomez, 2007; Edwards et al., 2010), recent reviews indicate that restoration efforts continue to focus on a few taxa (Bostrom-Einarsson et al., 2020; Ferse, Hein & Rölfer, 2021). Broadening the range of taxa used during propagation and outplanting is essential to ensure future coral reefs are functionally diverse and resilient to a range of stressors.

On the northern Great Barrier Reef (GBR), a research partnership with Tourism Operators and Traditional Owners has conducted extensive coral propagation and outplanting at high-value reef sites as part of the Coral Nurture Program (CNP). Historically, propagation and outplanting interventions developed within CNP have been applied in areas with good to moderate coral cover (10–50%; Howlett et al., 2021; Roper et al., 2022). Whether CNP methods are suitable at degraded or low hard coral cover systems (<10% hard coral cover) remain untested. For example, there have been minimal assessments of restoration at inshore sites along the GBR, particularly after such large disturbances as Tropical Cyclone Jasper. Inshore sites along the GBR are subject to relatively poor water quality from run-off and land-use (MacNeil et al., 2019; Petus et al., 2016), and have also been impacted by tropical cyclones (Puotinen et al., 2020), which has resulted in losses of hard coral cover and increases in macroalgal and turf algal cover. Following multiple disturbance events between 2002 and 2016 (*e.g.*, Tropical Cyclones), hard coral cover at inshore reefs declined by 41.5% at Palm Islands, 45.0% at Magnetic Island, 55.0% at the Whitsunday Islands and 45.6% at Keppel Island (Ceccarelli et al., 2020; Smith et al., 2022; Williamson et al., 2019). The recovery of these reefs is further challenged by factors including increased nutrient loads (De’ath & Fabricius, 2010; DeCarlo et al., 2020), reduced grazing intensity (Butler et al., 2024; Smith, Hunter & Smith, 2010) and increased competition with algae (Cheal et al., 2010; Smith et al., 2023). Additionally, turf algae can weaken and kill neighboring corals as they are quick to grow and are less vulnerable to grazing and water turbulence. Feedback mechanisms can perpetuate high levels of macroalgae and turfing algae, preventing coral recovery and leading to a loss of reef resilience (De’ath & Fabricius, 2010; Fulton et al., 2019; Johns et al., 2018), yet the efficacy of applying common coral propagation and outplanting techniques at these sites is unknown. Coral reefs dominated by algae and other non-coral organisms are likely to become more common in the future due to ongoing climate change. Consequently, it is essential we develop and optimise approaches to support recovery trajectories, enhance resilience and maintain key ecological functions and ecosystem services within these altered reef systems

Coral reefs can be slow to recover after significant mortality events because of large shifts in abiotic and biotic conditions. Contact competition between coral and algae (macroalgae and turf algae) can be a key factor to coral mortality (Clements et al., 2018; Lirman, 2001; McCook, Jompa & Diaz-Pulido, 2001) and may impact natural recovery at degraded or algal-dominated sites. This competition can reduce available space (Barott et al., 2012), overgrow neighbouring coral, and suppress growth and survivorship (Barott et al., 2012; Jompa & McCook, 2003), with varying differences depending on coral morphology (Swierts & Vermeij, 2016). Faster growing corals (*i.e.,* branching) grow faster to avoid benthic competition while slow growing corals (*i.e.,* massive and encrusting) have defensive mechanisms to avoid contact competition (Swierts & Vermeij, 2016). Coral size can also influence susceptibility to algal growth, outplant growth and survivorship. Coral-algal competition primarily impacts smaller corals (between 2–5 cm) and/or outplanted corals that can be overgrown by faster-growing algae (Lirman, 2001; Lirman & Biber, 2000; Yap & Molina, 2003). While small outplants (*e.g.*, microfragments) typically have higher growth rates than larger outplants (Page, Muller & Vaughan, 2018), larger outplants typically have greater survivorship (Smith & Hughes, 1999). Further, competition with algae (*e.g.*, *Dictyota*) has been recognised as a key driver affecting the survivorship of outplanted *Acropora cervicornis* in Florida where the highest survivorship was recorded in areas with <15% macroalgae cover (Van Woesik, Ripple & Miller, 2018). As such, removing macroalgae and prioritising larger outplants has been recommended to increase survivorship of outplanted corals (*e.g.*, Florida; Serafy et al., 2013) and to increase natural coral larvae settlement and recruitment (*e.g.*, Magnetic Island; Smith, Hunter & Smith, 2010). However, low overall survival was recorded for branching and bushy coral species outplanted after turf algae removal at three degraded inner shore sites in the Whitsundays (Scott et al., 2024a; Scott et al., 2024b). The low survival was suggested to primarily be due to coral dislodgement rather than competition with turf algae (Scott et al., 2024a; Scott et al., 2024b). As such, it remains unclear how site preparation influences success of outplanted corals at algal-dominated locations on the GBR and/or how this differs across diverse coral growth forms.

In this study, we evaluated how site preparation and outplant size impacted the survival and health of two coral species after outplanting at a highly degraded site on the northern GBR. Common outplanting techniques were applied and evaluated to determine their suitability in a highly degraded system. We hypothesised that (i) corals would have greater growth and survivorship in cleaned plots compared to uncleaned plots, (ii) that smaller outplants would have greater growth, while larger outplants would have greater survivorship and (iii) there would be species-specific suitability to outplanting at a degraded reef. Results from this study further our understanding of active intervention steps that can be taken by reef practitioners to improve assist natural recovery at highly degraded reefs sites on the GBR.

## Materials & Methods

### Study site

In September 2024, 16 outplanting plots were established at Low Isles reef on the Northern GBR, Australia (16°23′02.4″S 145° 33′35.5″E). Low Isles (Islets) are two islands located 15 km from Port Douglas in Far North Queensland. Low Isles has significant cultural, historical, ecological and economic value. The islands, also known as Wungkun, have significant cultural value to the Kuku Yalanji and Yirraganydji Traditional Owners whose Sea Country overlaps at Low Isles (Low Isles Douglas Shire Historical Society, 2025). Low Isles is also a high-value tourism site with thousands of visitors annually (Great Barrier Reef Marine Park Authority, 2025). In December 2023, a Category 2 Tropical Cyclone (TC Jasper) passed through Far North Queensland making landfall 110 km north of Low Isles, leading to heavy rainfall in the Low Isles Reef Catchment area (Emanuel, 2024). The Cairns/Port Douglas region recorded their highest daily rainfall in 100 years, with total rainfall during the event exceeding 2,000 mm (Emanuel, 2024). Low Isles was subsequently inundated with freshwater which caused high rates of coral mortality in the shallow reef flats, leading to an algal turf dominated state in the upper 3 m of the reef (P. Strudwick, 2024, per comm.).

### Experiment set-up

**Figure 1:**
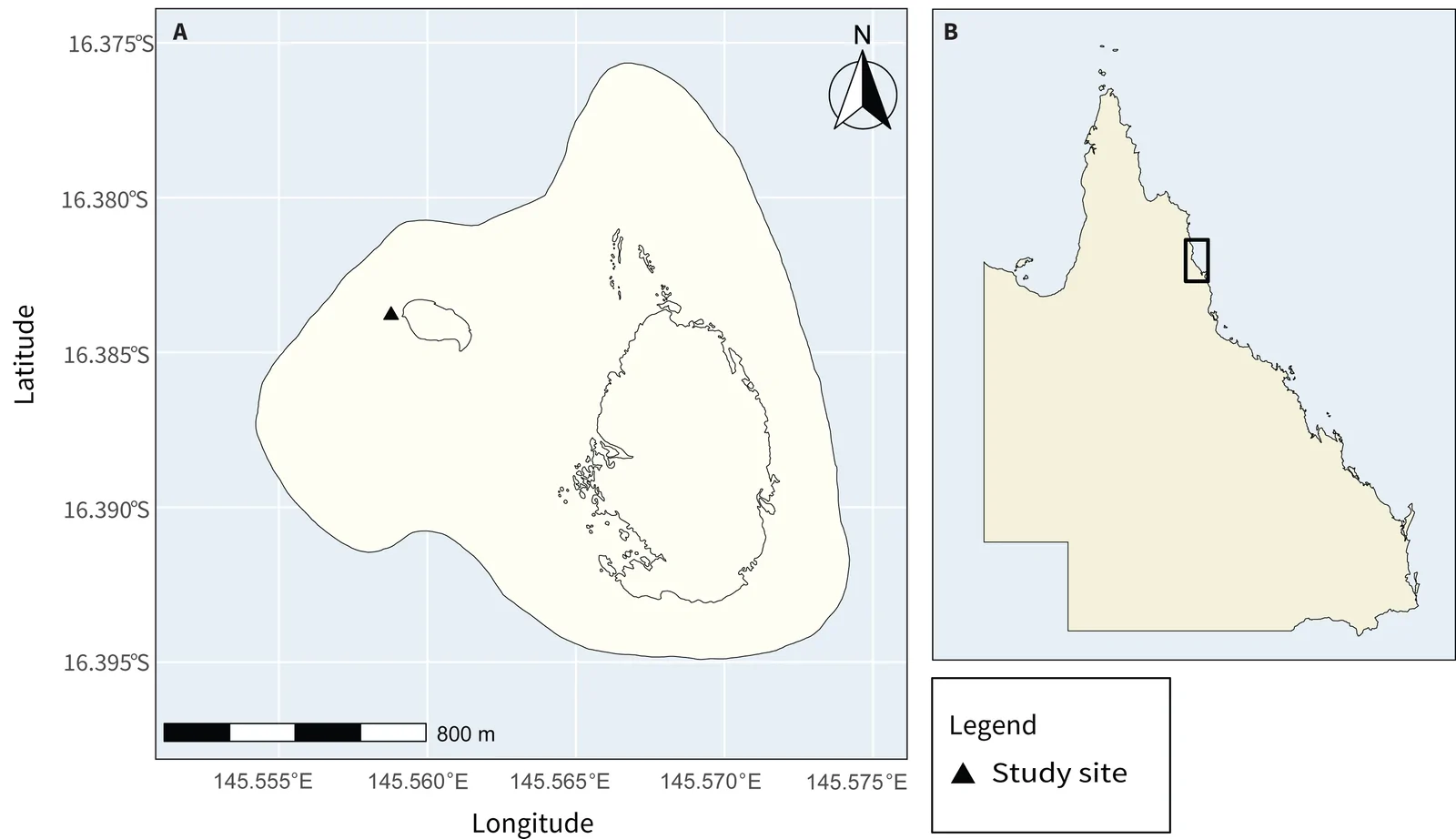
Map of study site in relation to Queensland, Australia. Map depicting (A) the location of the study site at Low Isles (triangle) and (B) Queensland, Australia, the location of Low Isles (black box). Map data ©2022, Allen Coral Atlas. Created using R Studio.

**Figure 2:**
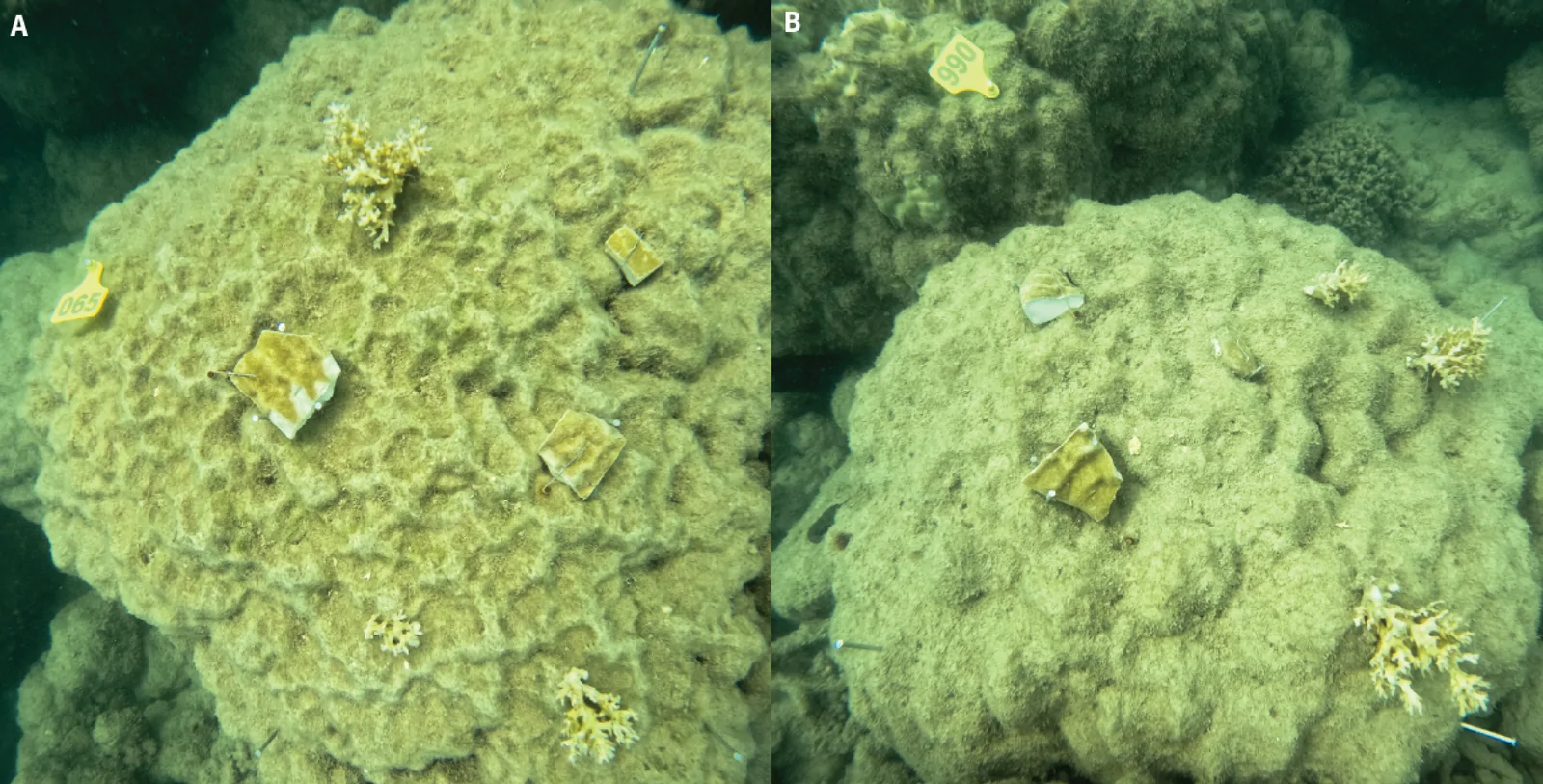
Top-down photographs of treatments. Top-down photographs of (A) cleaned plot and (B) uncleaned plot. Within each plot, two coral species, *Pocillopora acuta* and *Porites lutea,* were fragmented into three size classes (small: 2–5 cm, medium: 5 –10 cm and large <10 cm). Photo credit: Yi Da Jiang.

All activities were conducted under permit G23/48839.1 from the Great Barrier Reef Marine Park Authority. Sixteen plots (60 × 60 cm) at depths of 2–3 m were established between 6–9th September 2024 outside of the Locality Zone at Low Isles (16°23′4.19″S, 145°33′27.20″E; Fig. 1). Plots were established on bare substrates that included bare reef rock and dead massive corals (*e.g.*, *Porites* sp.). Plots were positioned in pairs of one cleaned and one uncleaned plot to minimise micro-environmental differences between the plots that could impact survivorship (*e.g.*, see Strudwick et al., 2023). Prior to outplanting, eight plots were cleaned of sediment and turfing algae with a soft grip scrub brush and eight plots remained uncleaned. Within each plot, six fragments of two coral species (*Pocillopora acuta* and *Porites lutea*) were planted (three outplants per species, detailed below; Fig. 2). Eight colonies of each species were collected from the Low Isles Reef (outside the Locality) at a depth of -4 m and two fragments of three size classes were harvested from each colony (six fragments in total from each colony): small (2–5 cm in length), medium (5–10 cm in length) and large (>10 cm in length; Fig. S1). Large fragments of *P. lutea* were collected with a hammer and chisel and were further fragmented ex- situ with a diamond blade mitre saw. During fragmentation with the saw, each colony was temporarily held in shaded buckets of fresh seawater for no more than 30 min. After fragmentation, the corals were rinsed twice to remove coral dust and mucus. *P. acuta* colonies were fragmented *in-situ* with a hammer and chisel. For both species, one fragment of each size class was haphazardly positioned within a cleaned and uncleaned plot and planted using a CoralClip^®^ (Suggett et al., 2020). For *P. lutea* fragments, 50 mm concrete nails were also placed around the fragment to secure larger fragments. All coral outplants were positioned 10–15 cm apart within the plot (see Figs. 2A and 2B).

### Monitoring

To track the health of outplanted corals, the plots were monitored at routine intervals over six months. Top-down photographs were taken from about one metre above (with an Olympus OM System TG-7) each plot to quantify paling of corals and to record mortality or coral dislodgement at six timepoints (T0, T3d, T8d, T40d, T73d and T170d after the start of the experiment). A coral health index card was placed in the plot when photographs were taken to provide a white reference for later quantification of coral paling (detailed below). Initial height, width and total length extension (TLE) measurements were collected for each coral outplant at T0 with a metal ruler.

### Benthic surveys and environmental data

To characterise the benthic composition of the study site, representative benthic images were taken with an Olympus OM System TG-7. A quadrat (60 × 60 cm) was placed at 50 equal intervals (every four fin kicks) on the substrate in a belt transect along a consistent depth (2–3 m). After image quality control (*e.g.*, removal of blurry images) a subset of 40 high quality images was used for subsequent benthic analysis. BioImage Indexing, Graphical Labelling and Exploration (BIIGLE) was used to process quadrat images from the belt transect survey. Images were uploaded to BIIGLE 2.0, an online open-source tool developed for marine images using customised tools for annotation and labelling. Machine Learning Assisted Image Annotation was used to conduct automated detection of objects. During the training phase, biotic (live/dead coral, invertebrates, algae) and abiotic (rubble, sand, rock) materials were manually annotated to enable accurate model learning and subsequent automated processing. Image segmentation and annotation adjustments were made manually when required. Substrates were categorised as macroalgae, abiotic (*i.e.,* rock, sand, rubble), invertebrates (clams), dead coral or live coral. All live Scleractinian corals were identified to genus level. Benthic composition was then converted to a percentage by first placing four laser points on the corner edges of the quadrat as a size reference within the images by utilising the DELPHI tool (Detection of Laser Point in Huge Image collections using Iterative learning). Percent cover of each category was then calculated by dividing the surface area of each component by the overall surface area of the quadrat (*e.g.*, X cm^2^ hard coral divided by 3,600 cm^2^ multiplied by 100 = percentage hard coral cover). Mean percent cover and standard deviation/error of each category was then calculated from the package *dplyr* (Wickham et al., 2023) in R Studio version 4.4.3 (R Core Team, 2025).

Supplementary sea surface temperature was obtained from the National Oceanic and Atmospheric Administration (NOAA) CoralTemp v3.1. global 5 km Satellite (NOAA, 2019). Data was retrieved from the Northern Great Barrier Reef satellite as it is the nearest satellite grid point to the study site. Data was extracted for the periods between 1^st^ August 2024 to 1^st^ March 2025.

To determine sediment characteristics at the study site, sediment pods were deployed for seven days at the start and end of the study. The pods were constructed with a Holman 100 mm polyvinyl chloride (PVC) coupling (Fig. S2). Four sediment pods were initially deployed from September 9th to September 16th 2024. However, the small amount of sediment collected was not adequate to determine particle size, therefore, the pods were redeployed from February 7th to February 25th 2025. The pods were selected as they provide a more accurate representation of sediment characteristics than traditional sediment tube traps since they allow for both the deposition and resuspension of sediments (Field, Chezar & Storlazzi, 2013). Sediment pods were collected *in-situ* by placing a screw cap onto the coupling to retain sediments and water. Sediments were flushed with freshwater, isolated in filters and transferred into 500 mL containers to be transported back to the University of Technology Sydney (UTS) for analysis. Sediment analysis was conducted at UTS, Sydney. Sediments were dried at 55 °C for 24 to 48 h, dry weighed (to the nearest 0.001g) and analysed for particle size distribution (Browne et al., 2012). Particle size was determined using a Malvern Mastersizer 2000 (Malvern Instruments Ltd, Worcestershire, UK) laser particle sizer for fine sediment. A dilution of 0.5% Calgon, a dispersing agent to break up particles, was used in 450 mL of reverse osmosis water to break down aggregates and disperse fine grain particles. Each pod was sampled three times to quantify mean particle size distribution. Particle size was determined *via* the Wentworth grain size classification chart (Wentworth, 1922). Wentworth size classes include sand (very fine sand 63 µm to very coarse sand 1000 µm), silt (very fine silt 3.9 µm to coarse silt 62 µm) and mud (<3.9 µm).

### Survivorship, coral health and growth rates

To determine survivorship, outplanted corals were recorded as alive (*i.e.,* alive fragments found in the CoralClip^®^), dead (*i.e.,* dead coral fragments still attached with CoralClip^®^ and covered in turfing algae) or dislodged (*i.e.,* CoralClips^®^ that were found empty) at each time interval (T0, T3d, T8d, T40d, T73d, T170d). The proportion of outplants in each category was calculated as a percentage of the total number originally outplanted. The proportion of algal growth to total outplant surface area (SA) was quantified at T170 using ImageJ. The polygon tool was used to trace algae found on the outplants and the planar surface area of the outplant. The proportion of algal cover was defined as the surface area of algae relative to the total outplant surface area, including live tissue and exposed skeleton. Change in algal cover over time was calculated by subtracting T0 values from T170 values, and were reported as Δ algae SA.

To determine changes in coral health, degree of coral ‘paleness’ was quantified using ImageJ for T0, T3d, T8d, T40d, T73d, T170d. Coral paleness is suggested to correlate with chlorophyll a and symbiont density and, hence is a non-invasive method to describe coral health (Amid et al., 2018; Chow et al., 2016). Images were converted from 24-bit colour to 8-bit colour, grayscale values were extracted from five (between 30 mm^2^–100 mm^2^) circles on each outplant. Colour was only quantified on live tissue areas; no part of the coral skeleton or shaded area was measured. An absolute white value was collected from each photograph to calculate the Mean Intensity Grey percentage (MIG%). The higher the percentage, the paler the coral. To determine changes, percentage change in MIG% was calculated by subtracting T0 values from each subsequent time point and values were reported as ΔMIG.

To quantify high resolution structural changes, outplanted corals were reconstructed using 3D modelling. Images were extracted from videos taken with an Olympus OM System TG-7 (4k 30 frames per second) of each plot at the first (T0) and at the final time point (T170d). For comprehensive coverage of each plot, a horizontal and vertical lawnmower technique was used to collect images and additional spirals were conducted for complex outplants. Six frames per second were extracted to Jpeg images from the 4K videos with Adobe Media Encoder. Three- dimensional models were reconstructed from images using AgiSoft Metashape Pro v 2.0 (AgiSoft Metashape Professional, 2024). Images were revised for quality using Estimate Image Check, where images rated below 0.5 were removed from analysis. Images with ratings below 0.5 (0 = lowest quality, 1 = highest quality) indicated that the quality of the image was too low to be reconstructed into three-dimensions. A batch script in Metashape Pro was run with the following settings: alignment = high accuracy and generic precision, 40k point limit, 4k tie point limit, adaptive camera model fitting, build dense cloud = medium quality, mild depth filtering, build texture = generic mapping model, texture from all cameras and hole filling enabled, build model = source data dense cloud with medium depth map quality. Nails that marked the plot boundary were flagged to determine the depths of each plot, and cattle tags (of known size) used to scale the model. The live tissue on each outplant was traced using the polygon selection tool in the orthomosaic layer. Changes in tissue SA (cm^2^) were calculated by subtracting T0 SA values from T170d values.

### Statistical analysis

All statistical analyses were conducted in R Studio version 4.4.3. (R Core Team, 2025). Data visualisations were generated in R using the package *ggplot2* (Wickham, 2016). Survivorship of outplanted corals was assessed with the application of pairwise log-rank tests on Kaplan–Meier survivorship probabilities (Lee & Wang, 2003). Survivorship probabilities were calculated from data that included corals that were dead or missing as ’events’ across plots at each time point. Survival probabilities of corals were calculated at T0, T3d, T7d, T40d, T73d and T170d, and compared to assess differences in survival probabilities across species, treatments (cleaned *vs* uncleaned), size classes and interactions between treatments and size classes. Functions utilized for survivorship analysis were drawn from packages: *survival* (Therneau, 2024) and *survminer* (Kassambara, Kosinski & Biecek, 2024). A one-way analysis of variance (ANOVA) was conducted to determine differences in the proportion of algae cover on *P. acuta* outplants and *P. lutea* outplants at T170d. Due to the low number of outplants remaining, outplants were pooled into species (as opposed to plot treatment or outplant size class). Parametric test assumptions were evaluated through the Shapiro–Wilk test for normality and visual inspection of residual plots. All assumptions were met to determine differences in the proportion of algae growth. A Kruskal–Wallis non-parametric one-way ANOVA was conducted to determine differences in coral tissue surface area change from T0 to T170d for *P. lutea* outplants at the end of the study.

Linear Mixed Models (LMM) were generated to assess the influence of site preparation (cleaned *vs* uncleaned) and outplant size (small, medium, large) on coral paleness over 170 days. All assumptions of linearity, homoscedasticity and residuals were met. The model was fitted using the lme function in the *lme4* package (Bates et al., 2015). Fixed effects included treatment, outplant size, species, and time. Random effects accounted for variation from plots number and coral outplant identity to control for repeated measures over time. Model simplification was conducted using ANOVA to compare models with progressively simplified fixed effects. *P*-values were obtained by likelihood ratio tests of the full model with the effect in question against the model without the effect in question.

## Results

### Environmental data

The benthic composition of Low Isles reef flat was comprised of live hard coral, dead coral, abiotic material, algae and invertebrates. Average hard coral cover (±standard error) at the site was 3.43 ± 1.38% (Fig. S3A) and the dominant genus was *Porites* (3.76 ± 1.66% of total hard coral cover), followed by *Galaxea* (0.99 ± 0.81% of total hard coral cover) and *Favites* (0.89 ± 0.25% of total hard coral cover). Abiotic material (*e.g.*, rock, rubble and sand) and dead coral were the primary benthic categories (20.89 ± 2.57% and 20.02 ± 3.40% cover respectively). Macroalgae and invertebrates were present in lower abundances (1.16 ± 0.32% and 0.32 ± 0.02% cover respectively). Notably, all rock substrate within the abiotic category was covered with turfing algae (Fig. S3B). Across the study site, sediments ranged from medium silt (15.6–31 µm) to medium sand (200–500 µm). Additionally, sea surface temperature retrieved from NOAA indicated an upward trend in sea surface temperature towards the end of the study (Fig. S3B) coinciding with the summer period.

### Coral survivorship

#### Survivorship differed across coral species but was consistent across treatments

**Figure 3:**
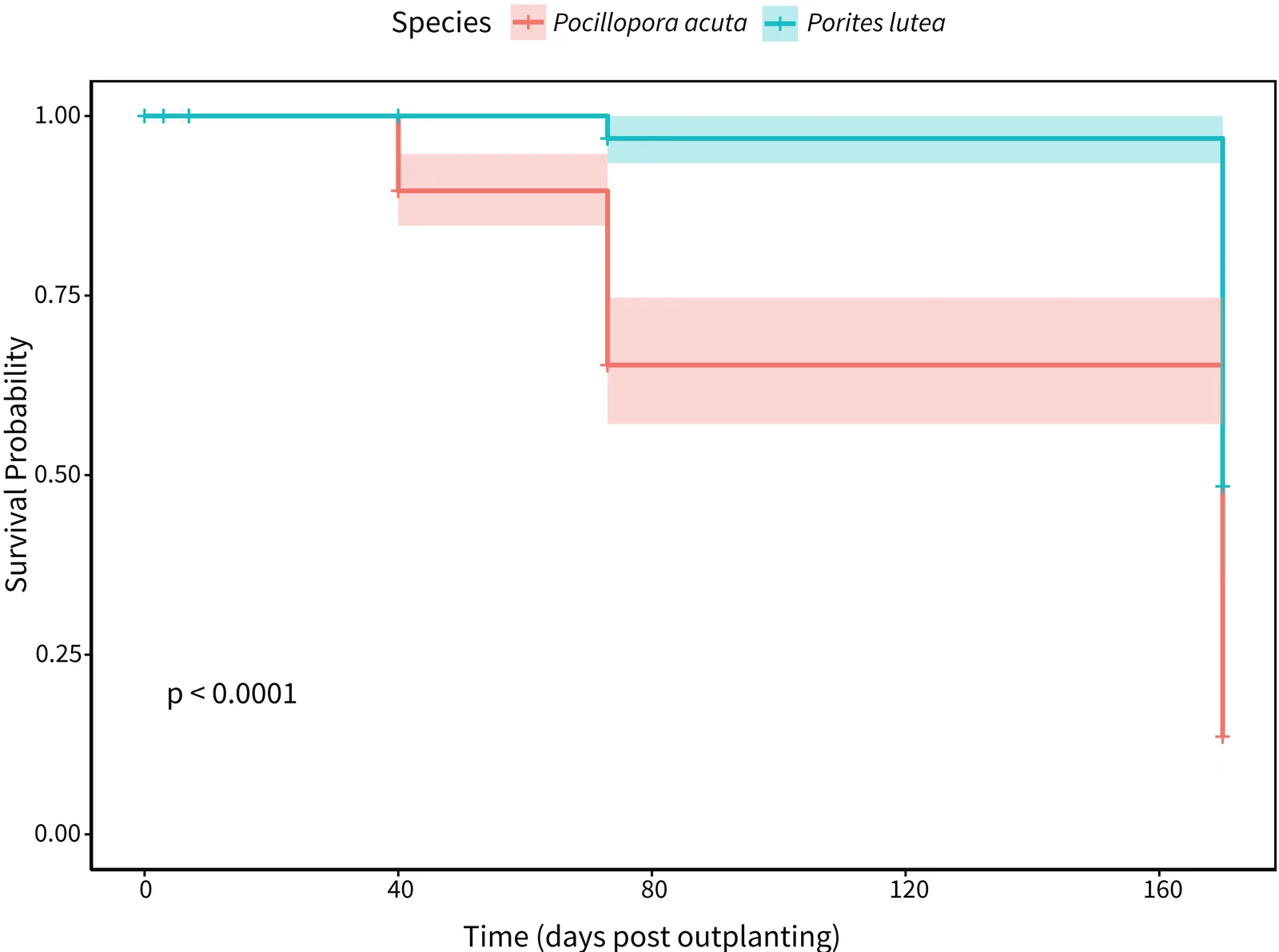
Kaplan–Meier survivorship probability for outplanted fragments of two species. Survivorship probability of *Pocillopora acuta* and *Porites lutea* at Low Isles over 170 days. Shaded lines indicated 95% confidence interval. *P*-values denotes significant difference between species.

The survival probabilities of the outplanted fragments after 170 days was higher for *P. lutea* (0.48 ± 0.07) compared to *P. acuta* (0.14 ± 0.04). Kaplan–Meier survival probability significantly differed between *P. lutea* and *P. acuta* (Pairwise log rank = 44.2, df = 1, *p* = 9 × 10–11; Fig. 3). When survival probabilities were examined in relation to site preparation after 170 days (for each species separately), there was no significant difference in survival probability between cleaned and uncleaned plots for *P. lutea* (0.46 ± 0.10 and 0.51 ± 0.10 respectively, Pairwise log rank = 0, df = 1, *p* = 1; Fig. S5A) or for *P. acuta* (0.12 ± 0.05 and 0.15 ± 0.06 respectively, Pairwise log rank = 0.6, df = 1, *p* = 0.4, Fig. S5B).

When survival probabilities were examined in relation to outplant size (for each species separately), there were no significant differences in survival probability for *P.lutea* outplants across size classes (small outplants = 0.36 ± 0.12, medium outplants = 0.53 ± 0.12, and large outplants = 0.56 ± 0.12; Pairwise log rank = 0.5, *df* = 2, *p* = 0.8; Figs. S5C & S5E). Similarly, there were no differences in survival probabilities for *P. acuta* outplants across size classes (small outplants = 0.08 ± 0.05, medium outplants = 0.12 ± 0.06, large outplants = 0.20 ± 0.08; Pairwise log rank = 0.3, *df* = 2, *p* = 0.8; Fig. S5D & S5F). There was no relationship between survival, site preparation and outplant size for either coral species (*P. lutea*: Pairwise log rank = 8.5, *df* = 5, *p* = 0.1, *P. acuta*: Pairwise log rank = 1.6, *df* = 5, *p* = 0.9; Table S1). Overall, survivorship differed between species, however, there were no treatment or size effects on success of outplanted fragments.

#### Coral survival and tissue cover

**Figure 4:**
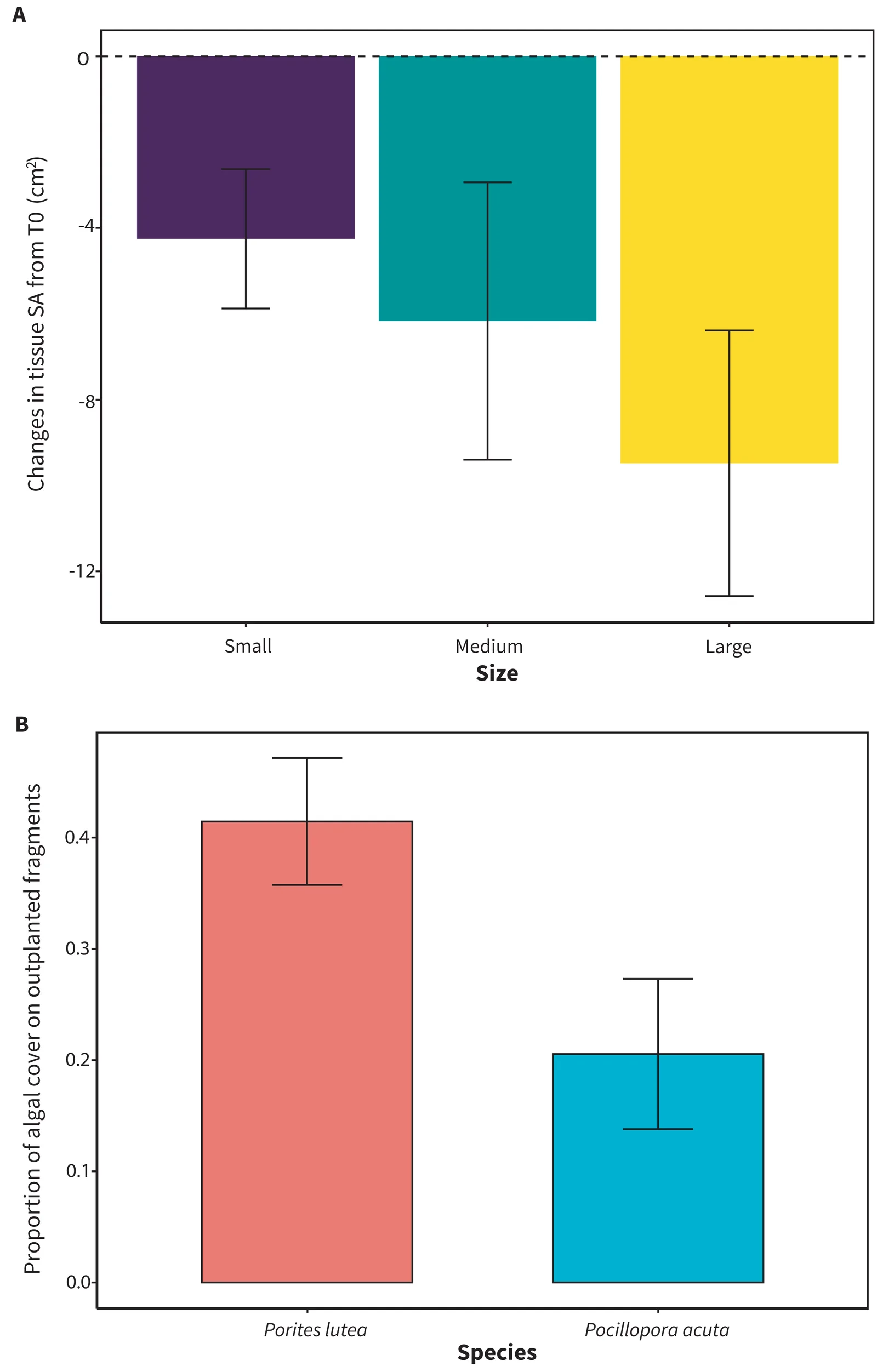
Changes in tissue surface area and proportion of algal growth on outplanted fragments. (A) Change in tissue surface area (cm^2^) for *P. lutea* outplants within three size classes 170 days after outplanting. Size categories are small (purple, *n* = 9), medium (green, *n* = 9) and large (yellow, *n* = 5). Error bars represent standard error, negative values indicate tissue recession. (B) Proportion of turf algal cover to total outplant surface area on remaining outplants at 170 days. All outplants were pooled into species: *Pocillopora acuta* (n =10) and *Porites lutea* (*n* = 24). Error bars represent standard error.

The proportion of alive, dead and dislodged outplants were calculated as a percentage of the total number originally outplanted. Survival probabilities were low for both species after 170 days with 44.83–77.08% ‘mortality’ for *P. acuta* and *P. lutea* respectively, however a large proportion of this loss was attributed to dislodged outplants (*P. acuta* 70.83 ± 6.56%, *P. lutea* 39.58 ± 7.06 % outplants dislodged *versus P. acuta* 6.25 ± 3.49%, *P. lutea* 6.25 ± 3.49% outplants dead, respectively; Table 1). Dislodgement was first observed at T40d, exclusively in *P. acuta* outplants (22.19 ± 6.07%) and by T73d, 4% of *P. lutea* and 45% of *P. acuta* outplants had been dislodged. Changes in tissue surface area from T0 to T170d was assessed for *P. lutea* outplants only as there were insufficient replicates for *P. acuta* due to high dislodgement by the end sampling period. There were no significant differences in tissue surface area for *P. lutea* between all outplanted size classes at the end of the study, and all size classes demonstrated tissue recession from tissue surface area at T0 (small outplants = −4.25 ± 1.62 cm, medium outplants = −6.17 ± 3.23 cm, and large outplants = −9.48 ± 3.09 cm; Kruskal–Wallis x2 = 0.61, *df* = 2, *p* = 0.74; Fig. 4A). Alongside tissue recession all *P. lutea* outplants exhibited turf algal cover, with up to 42.96 ± 6.1% on the outplanted fragments. The proportion of turf algal cover on the outplant (*i.e.,* SA of turf algal cover on outplanted fragment standardised to the SA of live tissue at 170 days) was calculated for the remaining outplants. Two outplants were excluded from analysis as the outplant boundary (where the coral fragment ended and the substrate began) could not be defined. Turf algal cover on coral outplants increased from 0% cover at T0 to 20.54% algal cover for *P. acuta* and 42.96% algal cover for *P. lutea* at T170d. *P. lutea* outplants had significantly greater turf algal cover (Δ algal SA 42.96 ± 5.70%) than *P. acuta* (Δ algal SA 20.54 ± 6.76%; F(1,32) = 0.31, *p* = 0.0424; Fig. 4B). Overall, low survivorship was primarily explained by corals that were unaccounted for (*e.g.*, dislodged from CoralClip^®^), with species-specific differences in turf algal cover recorded in the remaining outplants.

**Table 1:** Mean proportion (±standard error) of outplants status (alive, dead or dislodged) after 170 days. All outplant size and plot treatments are pooled under species (*P. lutea* and *P. acuta*). Alive refers to outplanted fragments found alive within the coral clip, dead refers to outplanted fragments that were covered in 100% algal cover and dislodged refers to an empty coral clip within the plot (counted as dead).

	***Pocillopora acuta***	***Porites lutea***
% corals alive (± standard error)	22.92 ± 6.07	54.17 ± 7.19
% corals dead (± standard error)	6.25 ± 3.49	6.25 ± 3.49
% corals dislodged (± standard error)	70.83 ± 6.56	39.58 ± 7.06

#### Coral health–paleness

**Figure 5:**
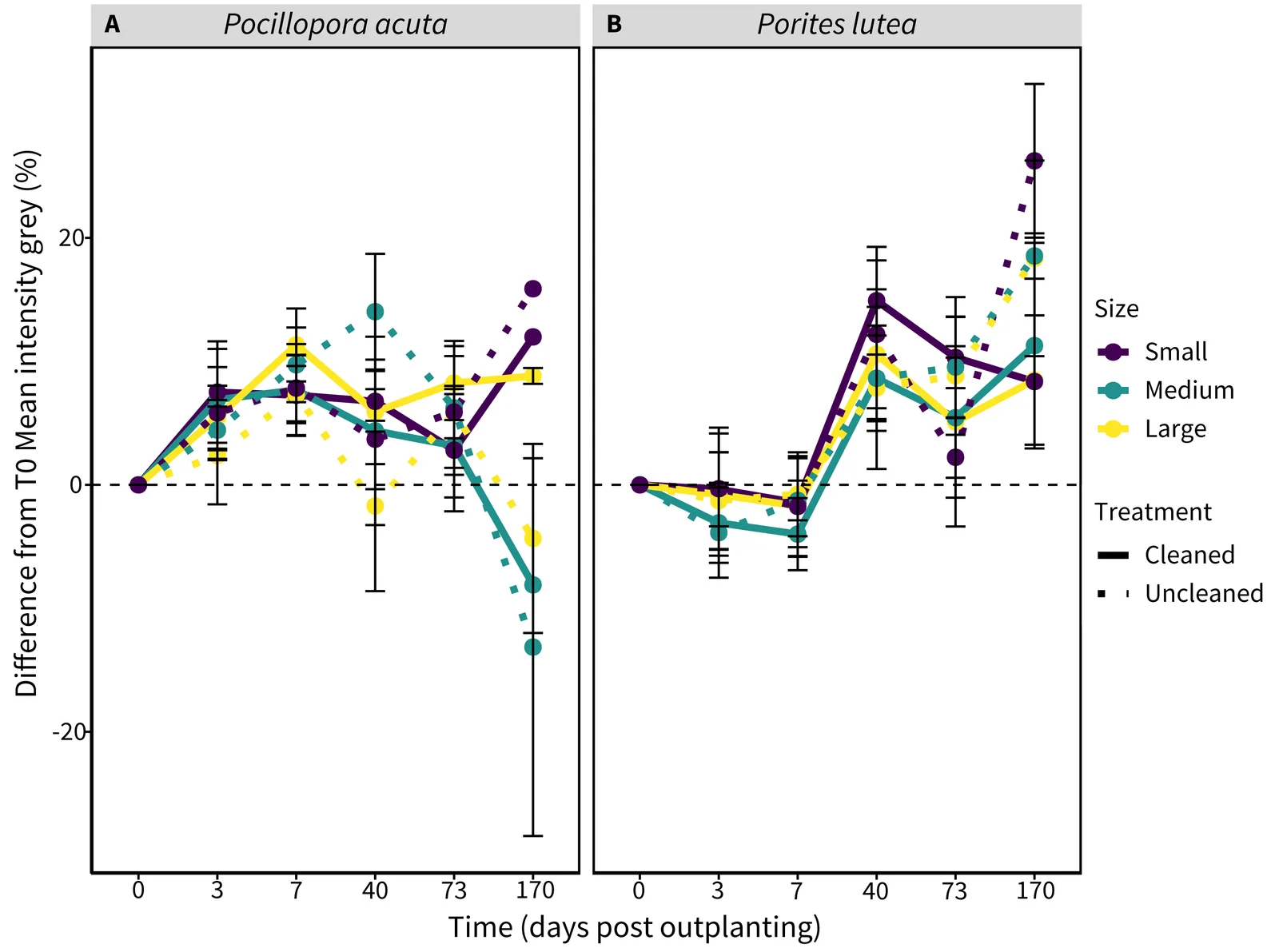
Differences in mean intensity grey percentage (MIG%) from T0 in two coral species. (A) *Pocillopora acuta* and (B) *Porites lutea* Size categories are small (purple), medium (green) and large (yellow). Cleaned plots represented by solid line and uncleaned plots represented by dotted lines. Dotted lines across 0 represent difference from T0. Error bars represent standard error (SE).

Time and species predicted differences in coral paling (positive or negative difference in MIG from T0, ± ΔMIG) (Model 3: *r*^2^ = 0.45, *p* < 2e-16; Table S2). *P. acuta* became paler between T3d–T40d (T3: ΔMIG +7.51 ± 3.62%; *p* = 0.04, T7: ΔMIG +7.26 ± 3.62%; *p* = 0.047 and T40d: ΔMIG +7.49 3.76%; *p* = 0.048; Fig. 5A). However, after T40d, medium *P. acuta* outplants began to show signs of recovery (decreasing MIG, which is suggested to indicate increased symbiont density and chlorophyll a (T40: ΔMIG −5.16 ± 5.45%, T73: ΔMIG −1.50 ± .75%, T170: ΔMIG −20.07 ± 11.25%); Amid et al., 2018). In contrast, *P. lutea* outplants corals became darker from T0 - T40d (−ΔMIG), after which *P. lutea* outplants increased in paleness between T40d - T170d (T40d: ΔMIG +14.91 ± 4.10%; *p* < 0.001, T73d: ΔMIG +10.31 ± 4.10%; *p* = 0.013 and T170d: ΔMIG +26.27 ± 9.01%; *p* = 0.004; Fig. 5B).

## Discussion

Algal dominated coral reefs are becoming more prevalent due to compounding environmental stressors driven by climate change (Graham et al., 2013; Inagaki & Longo, 2024). How reef restoration approaches can be applied to assist recovery of coral reefs affected by acute stressors (*e.g.*, Tropical cyclones) resulting in low coral cover on the GBR remains relatively untested. Therefore, we evaluated the application of coral outplanting techniques at Low Isles Reef—an inshore northern GBR reef site recently degraded by the 2023 Tropical Cyclone Jasper. This study provides early insights into the effectiveness of applying a coral outplanting reef restoration approach at degraded reef site on the GBR. In this study, cleaning outplant areas did not improve health and survival of outplanted corals, indicating that either cleaning is not required. However, a more frequent regime of cleaning may have provided increased benefits, or other factors (*e.g.*, high nutrient load, turbidity) may have been more influential in determining outplanting success at Low Isles. Size classes did not influence the survival or health in either species, suggesting that local environmental conditions and external stressors may have outweighed any size-related advantages reported in previous studies (Knoester et al., 2024; Lirman, 2001; Serafy et al., 2013). *P. acuta* outplants showed a decrease in paleness toward the end of the study, indicating an increase in symbiont density and chlorophyll a (Amid et al., 2018). *P. lutea* outplants exhibited greater survival than *P. acuta*, suggesting greater suitability of this coral species to outplanting at this degraded site. However, high proportions of outplants became dislodged and contributed to the low survival rates, which may have been due to fish predation (Horoszowski-Fridman et al., 2015; Koval et al., 2020), as there was high fish activity observed around the outplanted corals during survey times in this study. Challenges associated with attachment in this study may have further increased dislodgement and susceptibility to predation, thereby influencing observed outcomes. Despite greater survival, *P. lutea* outplants exhibited tissue recession and algal growth, indicating that competitive interactions with algae may inhibit growth of outplanted corals when outplanted with exposed skeleton. As such, we demonstrate the viability of outplanting corals at heavily degraded sites but highlight that differences in species’ suitability to outplanting and attachment methods should be carefully considered when applying such interventions in the future. However, long-term monitoring is required to confirm the long-term viability of the approach, including the survival, growth, reproduction and establishment of outplanted coral fragments.

### Cleaning outplant areas did not increase survival and significant algal growth occurred on outplants

In this study, cleaning outplant sites of sediment and turfing algae did not significantly increase health and/or survival of coral outplants. These findings are similar to other studies where the repeated removal of turfing algae (and associated biofouling organisms) on coral nurseries had not increased coral growth or changes in live coral tissue cover compared to where coral nurseries had minimal removal of turfing algae (Knoester et al., 2024). This contrasts with previous studies where the repeated removal of macroalgae has led to increased coral survival compared to areas where macroalgae was not removed (40% *versus* 29% survival respectively, Smith et al., 2023). The plots in this study were only cleaned once (prior to initial outplanting) and hence coral survival may have increased with additional cleaning. Further, the type of algae removed was fleshy macroalage (*e.g.*, *Sargassum* spp.) which could explain differences with Smith et al. (2023). Macroalgae and turfing algal growth on coral can limit the outplants’ ability to self-attach onto the substrate, thereby increasing the chance of physical dislodgement through strong water movement (Gomez, Dizon & Edwards, 2010), fish activity and/or predation (Koval et al., 2020; Rivas et al., 2021). Further, coral tissue growth can be hindered by turfing algae outcompeting or smothering the coral (Jompa & McCook, 2003; Lirman, 2001). Our findings are consistent with ex-situ trials between attachment methods (nails and cable tiles *vs.* cement) which found corals secured with nails exhibited low survival and algal growth, whereas outplants attached with cement had greater success and less algal growth (Unsworth et al., 2021). An alternative attachment method such as chemical adhesives (*e.g.*, cement) could reduce the amount of exposed skeleton, particularly for massive fragments, and thus reduce competition against turf algae growing on the coral skeleton. As such, future research should evaluate whether different cleaning regimes and adhesives such as cement (Unsworth et al., 2021) or epoxy (Williams & Miller, 2010; Forrester et al., 2011) can increase the success of outplanting at degraded sites. Overall, survival, growth and health of the outplants in this study were likely challenged by turfing algal growth, particularly on the exposed skeleton of *P. lutea* and on the base of *P. acuta*.

### Low survivorship may be driven by high outplant dislodgement rates

Outplant success rate at Low Isles was lower compared to other CNP reef sites (∼80% success; Howlett et al., 2021; Howlett et al., 2023) which in this study is primarily attributed to coral dislodgement. High coral outplant retention rates have previously been documented in the early stages of outplanting for the Coralclip^®^ in the Cairns/Port Douglas region (93–98% retention, 4–7 months post outplanting; (Suggett et al., 2020). However, the CoralClip^®^ has primarily been tested on branching and bushy morphologies at sites with higher coral cover (Howlett et al., 2023; Scott et al., 2024a; Scott et al., 2024b; Suggett et al., 2020), suggesting that an alternative attachment method may be more suitable for planting diverse morphologies in degraded systems where they may be exposed to a disproportionate amount of corallivory (Paige Strudwick, pers. obs.). In areas with low hard coral cover like Low Isles (<5% hard coral cover), fish may have been attracted to the new food source the outplants provided (Horoszowski-Fridman et al., 2015; Koval et al., 2020; Miller & Hay, 1998; Page, Muller & Vaughan, 2018). Constructing barriers with cages and metal spikes around coral outplants can effectively mitigate fish predation and could be considered for future outplanting efforts at Low Isles (Rivas et al., 2021). Additionally, using chemical adhesives to secure coral outplants would ensure the coral tissue is flush with the cement, which may reduce fish predation on the live tissue or dislodgement of outplants (Koval et al., 2020; Rivas et al., 2021). As such, we suggest other attachment methods should be tested to optimise retention of diverse growth forms in degraded systems. Consequently, future restoration efforts in degraded systems may consider the concurrent use of chemical adhesives and predation deterrents to increase the survival of outplanted corals.

### Broad environmental conditions likely outweighed fine-scale differences in outplanting environment from initial plot preparation

Environmental factors including high nutrient loads, increased algal dominance, and increased sedimentation can impact restoration success in degraded systems (Foo & Asner, 2021; Jessen et al., 2013; Roth et al., 2018). Inshore reefs are highly susceptible to increases in sedimentation from terrestrial run-off (Brown et al., 2017), with fine silt and clay shown to travel more than 3–5 km from the mainland (Carlson, Foo & Asner, 2019). In our study, no differences in coral health and survival were observed between cleaned and uncleaned plots, suggesting outplants were exposed to similar broad scale environmental conditions that had more influence that initial plot preparation. Further, at Low Isles the sediment was made up of silt and medium sand which can settle slowly (Erftemeijer et al., 2012; Storlazzi, Norris & Rosenberger, 2015), is susceptible to resuspension, and may have returned to plots shortly after cleaning thus returning them to a similar state as the uncleaned plots. Susceptibility of these sediment types to resuspension can cause challenging conditions for corals by reducing light availability for long durations and subsequently inhibiting coral growth (Storlazzi, Norris & Rosenberger, 2015) and may have exhibited a greater influence on the corals than plot preparation. When selecting a species for restoration in a degraded system, species-specific traits such as tolerance to high turbidity environments and/or susceptibility to disease should be considered (Madin et al., 2023). Tailoring outplanting approaches to consider potential spatial differences in broad scale environmental parameters including nutrient levels, salinity and turbidity at degraded sites and targeting species with inherent resilience to such conditions may help to increase survival and health of outplanted corals. In addition to these local stressors, elevated sea surface temperatures during then end of the experimental period may have further compromised coral health and survival. As increases in sea surface temperatures are projected to continue (Ruela et al., 2020; Masson-Delmotte et al., 2021) evaluating the effects of temperature stress is important and warrants further investigation in future studies.

### Species-specific traits may increase survival probabilities for coral outplants

*P. lutea* exhibited overall higher survivorship compared to *P. acuta* which may be related to species-specific traits that enable tolerance to certain environmental conditions. For example, some coral species have developed either morphological or physiological adaptions to withstand high sedimentation and turbid environments (Browne, Smithers & Perry, 2012). *P. lutea* and other *Porites* spp. secrete a protective mucus sheet that traps sediments but is easily sloughed off by waves and currents due to their smooth surface (Bessell-Browne et al., 2017; Smithers, Hopley & Parnell, 2006; Stafford-Smith & Ormond, 1992). Other tolerance mechanisms include switching to more heterotrophic feeding (*e.g.*, *Goniastrea*; (Anthony, 2000), tissue expansion (*e.g.*, *Platygyra*; Stafford-Smith & Ormond, 1992) and projection of polyps above the colony surface to modify their boundary layers (*e.g.*, *Goniopora*; Stafford-Smith & Ormond, 1992). These traits may explain why the dominant coral morphologies found in inshore reefs are massive morphologies on the GBR (Smithers, Hopley & Parnell, 2006). Future reef restoration efforts at degraded sites should consider selecting coral species with similar functional traits to *P. lutea*, such as *Goniastrea retiformis* or *Platygyra sinensis*, which have demonstrated tolerance to sedimentation and turbid conditions in other inshore reef environments (Anthony, 2000; Browne et al., 2014).

## Conclusion

As coral reefs continue to face compounding stressors, proactive interventions are becoming increasingly necessary to restore or retain coral reef services and values and to build resilience of these ecosystems to future environmental stressors. Our study examined foundational restoration approaches at an impacted reef and highlighted species-specific differences in health and survival of outplanted corals. *P. lutea* showed a greater suitability to outplanting at the site which we hypothesise to be due to an innate capacity to tolerate high sedimentation and turbidity (Fabricius, 2005) compared to *P. acuta*. Cleaning outplanting substrate did not align with increased survival or coral health in this study, however, more frequent cleaning schedules may provide greater benefits and should be explored. Additionally, we recommend that long-term studies should evaluate additional metrics of coral success including fecundity, calcification rates and heat resilience for outplanted corals in impacted systems to determine if and how outplanted corals are contributing to resilience of the ecosystem. Further, despite reduced coral outplant survival in this study (compared to previous CNP studies (see Howlett et al., 2023; Scott et al., 2024a; Scott et al., 2024b), it could be argued that any coral survival is relatively more valuable for the recovery of a degraded system, particularly if weighted against the low existing low coral cover. Our results suggest that reef restoration approaches may support natural recovery at heavily degraded sites and highlight the importance of tailoring restoration efforts to local environmental conditions.

## Supplemental Information

10.7717/peerj.21725/supp-1 Supplemental Information 1 Experimental design diagram Each colony was fragmented into six pieces (small: 2–5 cm, medium: 5–10 cm, large > 10 cm) and outplanted onto clean and uncleaned plots. Image credit: Created with BioRender.com

10.7717/peerj.21725/supp-2 Supplemental Information 2 Constructed sediment pod Sediment pod constructed with 100 mm Holman PVC coupling. Four marine grade stainless steel eyebolts (6 × 60 mm) were drilled into the side of the coupling to hold the concrete in place, to serve as handles and to mount the pods onto the reef. Cement was filled to cover the eye bolts (∼6 cm high) and left to dry with a rough surface to imitate coral surface. Photo credit: Yi Da Jiang.

10.7717/peerj.21725/supp-3 Supplemental Information 3 Environmental data of Low Isles (A) Bar plot showing the benthic composition of Low Isles reef flat (average percentage cover of categories). Benthic categories include abiotic material (rock, rubble and sand), dead coral, had coral, algae, and other invertebrates. Error bars represent standard error (SE). (B) Sea surface temperature obtained from NOAA CoralTemp v3.1 global 5 km satellite. Dates are between 1st August 2024 to 1st March 2025. Red dotted line indicates bleaching threshold temperature.

10.7717/peerj.21725/supp-4 Supplemental Information 4 An example of a representative benthic image Area within the quadrat (60 × 60 cm) was analysed to determine benthic characteristics. All dead corals were covered in turfing algae as seen in this example.

10.7717/peerj.21725/supp-5 Supplemental Information 5 Kaplan-Meier survivorship probability for outplanted corals in relation to site preparation and outplant size over 170 days Fates of (A) *Porites lutea* outplants and (B) *Pocillopora acuta* outplants. Size classes pooled together to determine fates in cleaned plots (red line) and uncleaned plots (blue line). Fates of outplant size classes split into small (yellow line), medium (green line) and large (purple line) in cleaned plots (C) *Porites lutea* outplants and (D) *Pocillopora acuta* outplants, and uncleaned plots (E) *Porites lutea* outplants and (F) *Pocillopora acuta* outplants. Shaded areas indicate 95% confidence intervals.

10.7717/peerj.21725/supp-6 Supplemental Information 6 Kaplan–Meier Survival probabilities outplanted coral at 170 days Survivorship probabilities were separated by species and size in relation to site preparation (cleaned *vs*. uncleaned) SE, standard error. CI, confidence interval.

10.7717/peerj.21725/supp-7 Supplemental Information 7 Linear mixed effects models on coral health (paleness) Linear mixed effects models for repeated measures with fixed and random effects. Fixed effects include difference in mean intensity grey values (MIG %) from T0: time, species, treatment (cleaned vs uncleaned) and size classes (small, medium, large). Random effects include outplant ID. Akaike information criterion (AIC) used to measure goodness of fit for each model. Lowest AIC represents better model fit.

## Additional Information and Declarations

### Field Study Permissions

The following information was supplied relating to field study approvals (i.e., approving body and any reference numbers): Field experiments were permitted under G23/48839.1 granted by the Great Barrier Reef Marine Park Authority.

### Data Availability

The following information was supplied regarding data availability: The data is available at Zenodo: Jiang, Y. D., Camp, E., Cobleigh, K., Roper, C.& Strudwick, P. (2026). Evaluating impacts of site preparation and fragment size on coral outplanting success at a highly degraded reef site [Dataset]. Zenodo. https://doi.org/10.5281/zenodo.17693306.
