# Supplementary material for: Evaluating the impacts of site preparation and fragment size on coral outplanting success at a highly degraded reef site
Source: PeerJ. 2026 Sep 14;14:e21725. doi: 10.7717/peerj.21725 (PMC13584934; doi:10.7717/peerj.21725)
Supplement: Supplementary file 1 [file peerj-14-21725-s001.pdf]

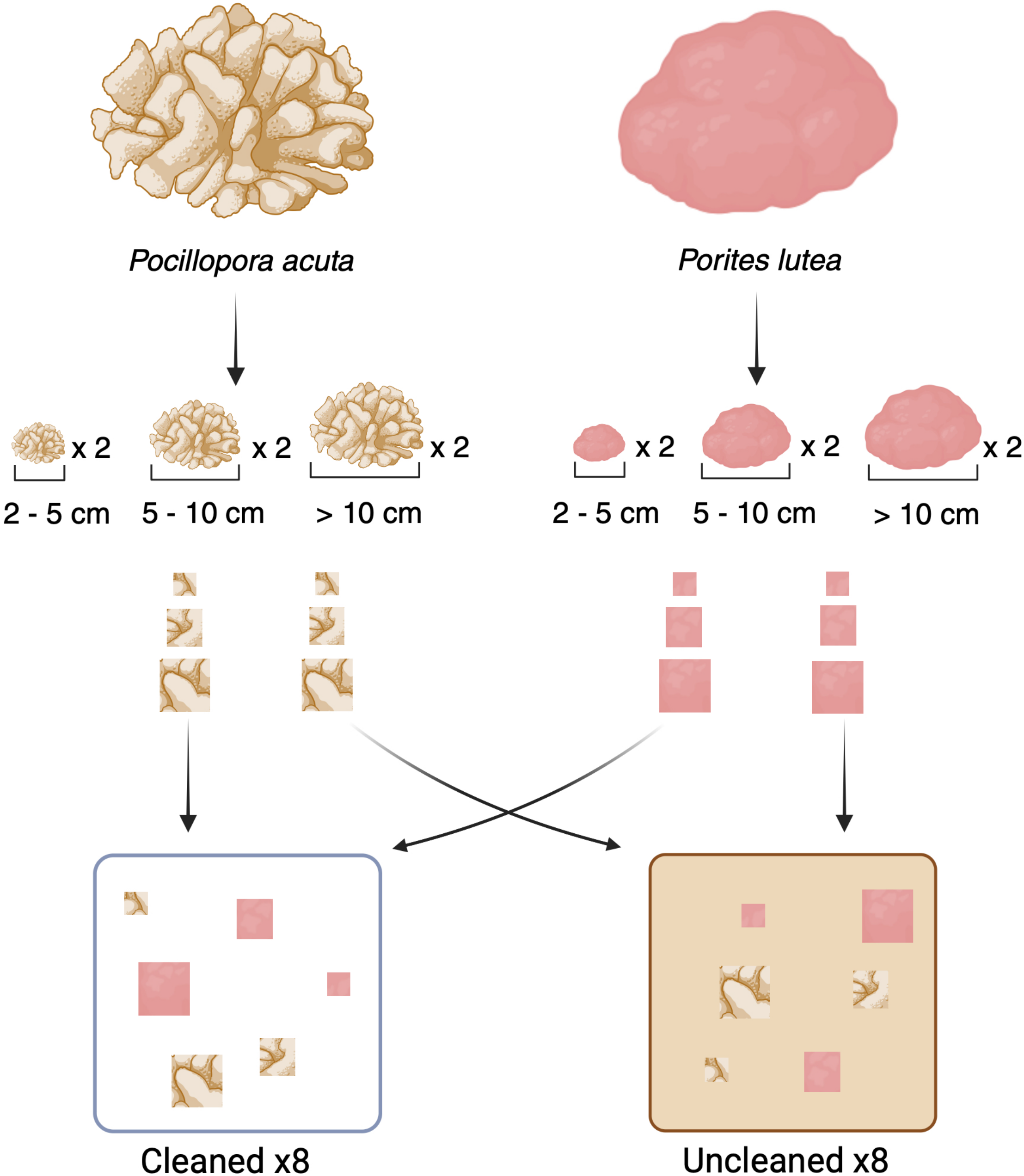

Pocillopora acuta
Porites lutea
x 2
x 2
x 2
x 2
x 2
x 2
2 - 5 cm
5 - 10 cm
> 10 cm
2 - 5 cm
5 - 10 cm
> 10 cm
Cleaned x8
Uncleaned x8
