# Supplementary material for: Evaluating the impacts of site preparation and fragment size on coral outplanting success at a highly degraded reef site
Source: PeerJ. 2026 Sep 14;14:e21725. doi: 10.7717/peerj.21725 (PMC13584934; doi:10.7717/peerj.21725)
Supplement: Supplementary file 2 [file peerj-14-21725-s003.pdf]

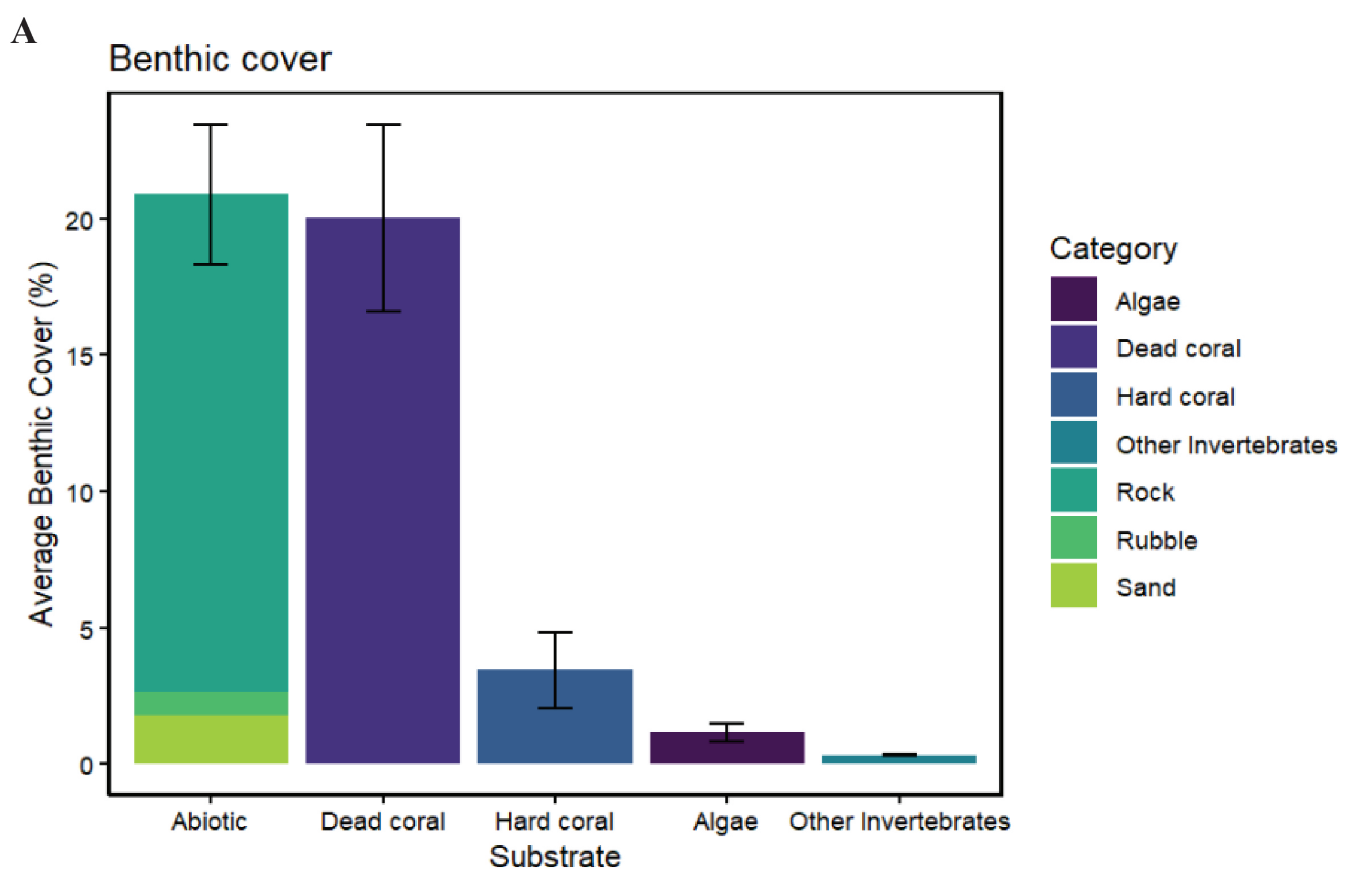
A
Benthic cover
Average Benthic Cover (%)
0
5
10
15
20
Abiotic
Dead coral
Hard coral
Algae
Other Invertebrates
Substrate
Category
Algae
Dead coral
Hard coral
Other Invertebrates
Rock
Rubble
Sand

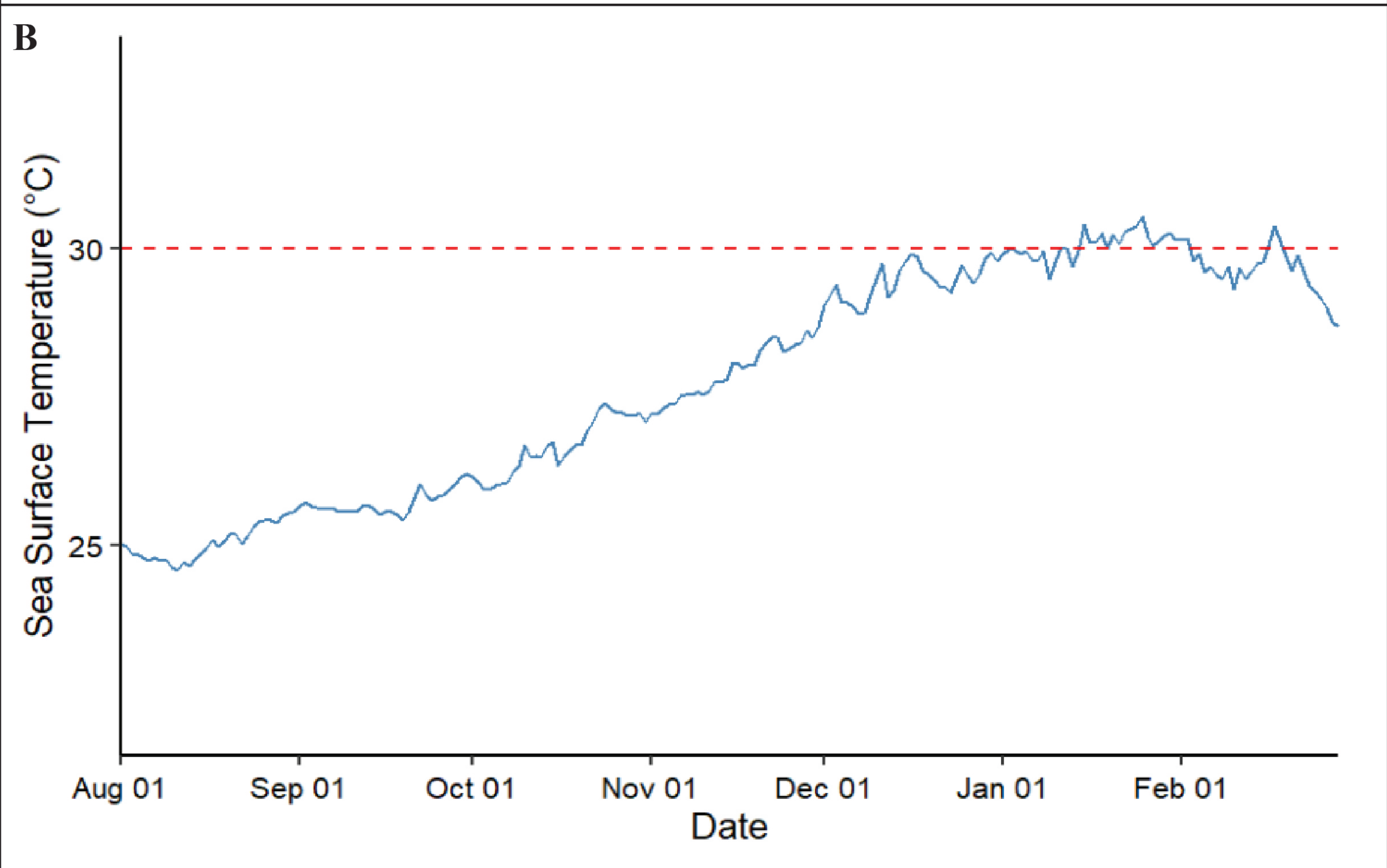
B
Sea Surface Temperature (°C)
25
30
Aug 01
Sep 01
Oct 01
Nov 01
Dec 01
Jan 01
Feb 01
Date
