# Supplementary material for: Evaluating the impacts of site preparation and fragment size on coral outplanting success at a highly degraded reef site
Source: PeerJ. 2026 Sep 14;14:e21725. doi: 10.7717/peerj.21725 (PMC13584934; doi:10.7717/peerj.21725)
Supplement: Supplementary file 3 [file peerj-14-21725-s005.pdf]

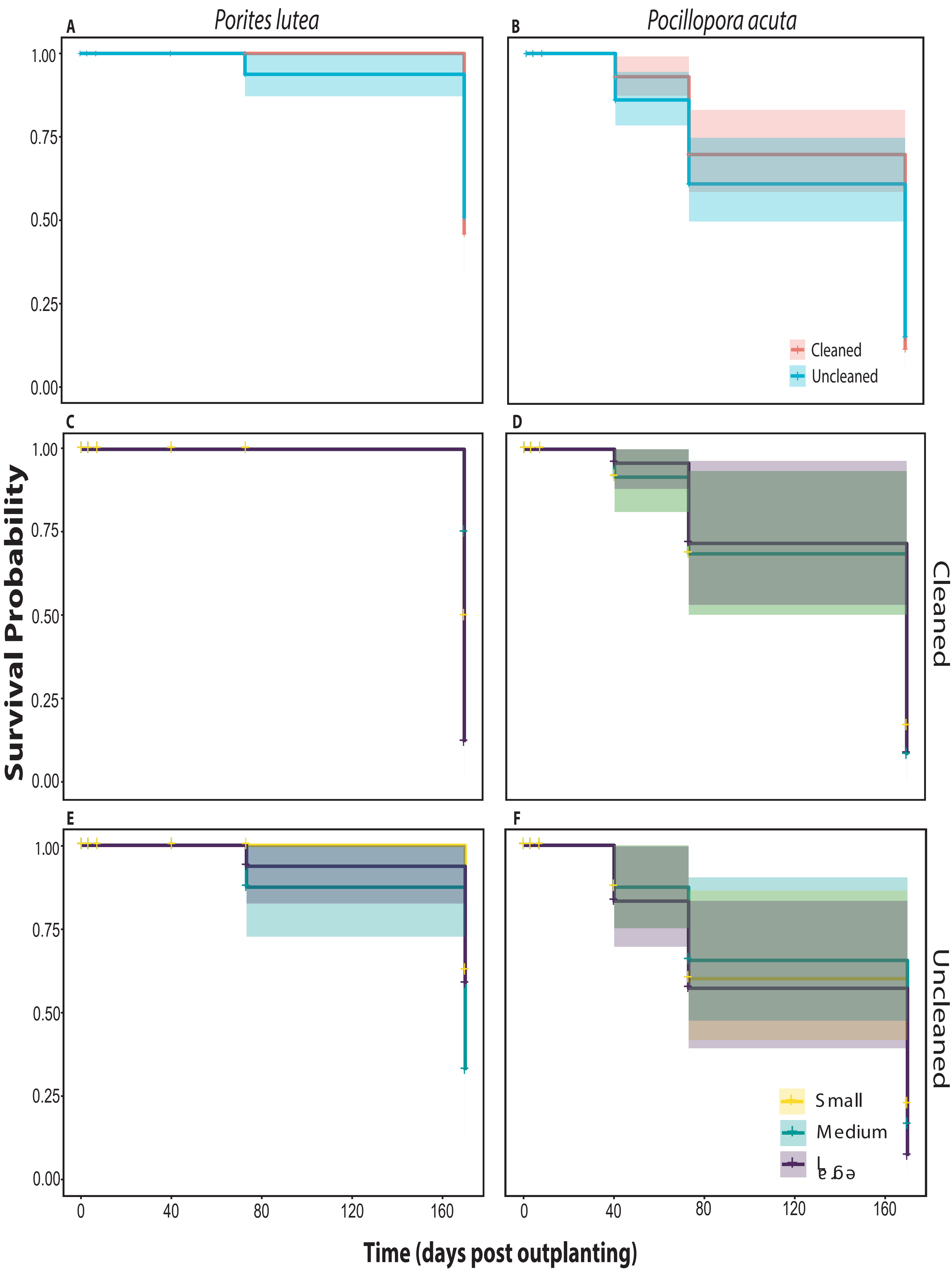
A
Porites lutea
B
Pocillopora acuta
C
D
E
F
Survival Probability
1.00
0.75
0.50
0.25
0.00
Cleaned
Uncleaned
Small
Medium
0
40
80
120
160
Time (days post outplanting)
