# Supplementary material for: Evaluating the impacts of site preparation and fragment size on coral outplanting success at a highly degraded reef site
Source: PeerJ. 2026 Sep 14;14:e21725. doi: 10.7717/peerj.21725 (PMC13584934; doi:10.7717/peerj.21725)
Supplement: Supplementary file 4 [file peerj-14-21725-s006.docx]

| **Species and Treatment** | **Size** | **n.risk** | **n.event** | **Survival** | **SE** | **Lower 95% CI** | **Upper 95% CI** |
| --- | --- | --- | --- | --- | --- | --- | --- |
| *P.lutea*  - Cleaned | Small | 8 | 7 | 0.125 | 0.117 | 0.02 | 0.782 |
|  | Medium | 8 | 2 | 0.75 | 0.153 | 0.503 | 1 |
|  | Large | 8 | 4 | 0.5 | 0.177 | 0.25 | 1 |
| *P.lutea* – Uncleaned | Small | 8 | 3 | 0.586 | 0.165 | 0.338 | 1 |
|  | Medium | 8 | 5 | 0.328 | 0.153 | 0.132 | 0.818 |
|  | Large | 8 | 3 | 0.625 | 0.171 | 0.365 | 1 |
| *P.acuta*  - Cleaned | Small | 8 | 7 | 0.089 | 0.085 | 0.014 | 0.575 |
|  | Medium | 8 | 7 | 0.086 | 0.082 | 0.013 | 0.551 |
|  | Large | 8 | 6 | 0.172 | 0.109 | 0.05 | 0.593 |
| *P.acuta* – Uncleaned | Small | 8 | 7 | 0.072 | 0.068 | 0.011 | 0.465 |
|  | Medium | 8 | 6 | 0.164 | 0.104 | 0.047 | 0.568 |
|  | Large | 8 | 5 | 0.226 | 0.111 | 0.086 | 0.592 |
