# Supplementary material for: Evaluating the impacts of site preparation and fragment size on coral outplanting success at a highly degraded reef site
Source: PeerJ. 2026 Sep 14;14:e21725. doi: 10.7717/peerj.21725 (PMC13584934; doi:10.7717/peerj.21725)
Supplement: Supplementary file 5 [file peerj-14-21725-s007.docx]

| **Model** | **Variables** | **Akaike information criterion (AIC)** |
| --- | --- | --- |
| M1 | MIG ~ time * species * treatment * size * (1\| fragment_ID) | 3401.5 |
| M2 | MIG ~ time * species * treatment *(1\| fragment_ID) | 3344.1^a^ |
| M3 | MIG ~ time * species * (1\| fragment_ID) | 3330.3^a,b^ |
| M4 | MIG ~ time * size * (1\| fragment_ID) | 3454.2 |
| M5 | MIG ~ time * treatment * (1\| fragment_ID) | 3453.8 |
| Null | MIG ~ time * (1\| fragment_ID) | 3442.9 |

^a^Likelihood ratio test (LRT) compared with Null (*P < 2e-16*)
